# Androgen Receptor Gene Polymorphism, Aggression, and Reproduction in Tanzanian Foragers and Pastoralists

**DOI:** 10.1371/journal.pone.0136208

**Published:** 2015-08-20

**Authors:** Marina L. Butovskaya, Oleg E. Lazebny, Vasiliy A. Vasilyev, Daria A. Dronova, Dmitri V. Karelin, Audax Z. P. Mabulla, Dmitri V. Shibalev, Todd K. Shackelford, Bernhard Fink, Alexey P. Ryskov

**Affiliations:** 1 Department of Cross-Cultural Psychology and Human Ethology, Institute of Ethnology and Anthropology, Russian Academy of Sciences, Moscow, Russia; 2 Department of Evolutionary and Developmental Genetics, Koltzov Institute of Developmental Biology, Russian Academy of Sciences, Moscow, Russia; 3 Department of Genome Organization, Institute of Gene Biology, Russian Academy of Sciences, Moscow, Russia; 4 Biological Faculty, Moscow State University, Moscow, Russia; 5 Department of Archaeology, College of Arts and Social Sciences, University of Dar es Salaam, Dar es Salaam, Tanzania; 6 Department of Psychology, Oakland University, Rochester, Michigan, United States of America; 7 Institute of Psychology, University of Göttingen, Göttingen, Germany; State University of New York at Oneonta, UNITED STATES

## Abstract

The androgen receptor (*AR*) gene polymorphism in humans is linked to aggression and may also be linked to reproduction. Here we report associations between *AR* gene polymorphism and aggression and reproduction in two small-scale societies in northern Tanzania (Africa)—the Hadza (monogamous foragers) and the Datoga (polygynous pastoralists). We secured self-reports of aggression and assessed genetic polymorphism of the number of CAG repeats for the *AR* gene for 210 Hadza men and 229 Datoga men (aged 17–70 years). We conducted structural equation modeling to identify links between *AR* gene polymorphism, aggression, and number of children born, and included age and ethnicity as covariates. Fewer *AR* CAG repeats predicted greater aggression, and Datoga men reported more aggression than did Hadza men. In addition, aggression mediated the identified negative relationship between CAG repeats and number of children born.

## Introduction

Aggression in traditional and modern societies is sometimes deployed to acquire resources and, therefore, social status and reproductive opportunities [1–6]. Even in forager societies with marked egalitarianism, aggressive competition between men for access to women is substantial, with most homicides attributable to competition between men [7,8–11]. Differences in mating systems may account for some of the variation in aggression. In hunter-gatherer societies, such as the monogamous Hadza of Tanzania (Africa), men invest more in offspring than in small-scale pastoralist societies, such as the polygynous Datoga of Tanzania [12–14]. Polygyny and between-group aggression redirect men’s efforts from childcare toward investment in male-male relationships and the pursuit of additional mates [15]. When men participate in childcare, their testosterone (T) level decreases [15–18]. Muller et al. [19] found that, among the monogamous, high paternally investing Hadza, T levels were lower for fathers than for non-fathers. This effect was not observed among the polygynous, low paternally investing Datoga. These results were interpreted as corroborating the ‘challenge hypothesis’ [20], which posits that T facilitates reproductive effort, including investment in mate-seeking, at the expense of parenting effort. According to the challenge hypothesis, T promotes aggression when this is beneficial for reproduction, as it is, for example, in combat with rivals over access to women.

The effect of androgens, such as T, operates through stimulation of androgen receptors [21–23]. The androgen receptor (*AR*) gene contains a polymorphic and functional locus in exon 1, comprising two triplets (CAG and GGN). This locus supports a regulatory function that responds to T, with fewer CAG repeat clusters being more effective in transmitting the T signal [22]. Moreover, the length of the GGN repeat predicts circulating and free T in men [23]. These relationships have inspired research investigating associations of the *AR* gene polymorphism with personality traits and behavioral correlates, including antisocial behavior and aggression [24]. The results of these studies are equivocal. Some studies report an effect of CAG and GGN polymorphisms on impulsive personality traits [25,26] and violence [27], whereas other studies do not find links between *AR* polymorphism and personality traits [28]. Butovskaya et al. [29] investigated aggression among male Datoga pastoralists and identified negative associations of the number of *AR* CAG repeats with aggression, anger and hostility. Men with fewer *AR* CAG repeats reported more aggression and sired more children. A previous study of these relationships among Hadza men did not find these associations [30], with the failure to replicate attributed to differences in cultural attitudes about aggression in the Datoga and Hadza. Butovskaya et al. [30] assessed men in a single culture, leaving open the possibility of culture-specific links between *AR* gene polymorphism and aggression.

Here we report associations between *AR* gene polymorphism and aggression and reproduction in men living in one of two small-scale societies, the Hadza and the Datoga of Tanzania. The Hadza are hunter-gatherers that are egalitarian and monogamous and have nominal leadership with no clear division into age classes [15,31–34]. Conflicts are typically resolved by separating the parties for a period of time and there is no special judicial institution for policing in-group violence [1,2]. Hadza men compete in the form of successful hunting, and female mate choice is important [35,36]. Consequently, male reproduction is positively associated with hunting skills and informal leadership [37]. The Datoga are seminomadic pastoralists [38], polygynous, and horizontally divided into generation sets with clear wealth stratification [39,40]. As in other polygynous societies [41–43], the social status and number of wives and children sired by a man are correlated with his wealth [40]. To address violence within families or clans, the Datoga have developed judicial institutions based on customary laws [38] that include public assembly, clan moots, and women’s and neighborhood councils. Using a system of fines and ostracizing of habitual aggressors, the Datoga manage within-tribal violence [44].

We predicted differences between Hadza men and Datoga men in aggression and in CAG repeats of the *AR* gene, due to differences in mating systems and related cultural differences in attitudes towards aggression. Manning [45] suggests that polygyny facilitates high T production and that high prenatal T facilitates the development of traits that support male competitiveness. Thus we hypothesized greater aggression and fewer and more variable CAG repeats in the polygynous Datoga than in the monogamous Hadza. We further hypothesized that more aggressive men have greater reproduction (number of children born). Also, men’s age is likely to positively predict the number of children born [46], and men reproducing in later age are subjected to lower mortality risk [47]. Finally, we hypothesized the relationships between CAG repeat number, age, and ethnicity with the number of children sired to be mediated by aggression.

## Materials and Methods

### Study samples

We studied 210 Hadza men and 229 Datoga men (aged 17–70 years) from northern Tanzania, Africa. Self-reports of aggression and buccal epithelium samples (for genotyping) were collected between 2007 and 2013 as part of a larger study. We secured age by using a calendar of well-dated and memorable events in local history, as the majority of Hadza and Datoga are not literate and do not keep birth records. Each participant was assigned to a 10-year age group (1 = 17–19 years; 2 = 20–29; 3 = 30–39; 4 = 40–49; 5 = 50–59; 6 = 60+ years) following previous protocols (e.g., [48]).

### Instruments

Participants were interviewed in Swahili by the first author or a trained local assistant. They were asked to provide information including their age, sex, marital status, number of children, ethnicity and aggression history (especially fights with other tribal members). All questions were read aloud in one-to-one dialogues and further explanations were provided, if necessary. Self-reported aggression was assessed with the Buss-Perry Aggression Questionnaire (BPAQ; [48]). The BPAQ includes 29 statements, grouped into four subscales—physical aggression (9 items), verbal aggression (5 items), anger (7 items), and hostility (8 items)—answered on a Likert scale anchored by 1 (*extremely uncharacteristic of me*) and 5 (*extremely characteristic of me*). The translation of the BPAQ into Swahili (S1 File) was done by one of the authors (A.M.), following accepted standards (translation and back translations by four bilingual assistants [49,50]. To ensure that participants understood the questions, the local assistant provided examples of actions related to the trait common in the culture. Scores on individual subscales and total BPAQ scores were calculated only for respondents who answered all items. Cronbach’s alpha for the total score was 0.77 for both the Hadza (*n* = 204) and Datoga (*n* = 190).

### Genotyping

Buccal epithelium samples were collected for DNA analysis. Genomic DNA of Hadza (*n* = 166) and Datoga (*n* = 114) was isolated using the Diatom DNA Prep 200 extraction kit (IsoGene Lab, Russia). The *AR* PCR was performed with the primers, 5′–(FAM)tccagagcgtgcgcgaagtgat–3′ and 5′–(FAM)cgactgcggctgtgaaggttg–3′. The reaction mixture was as described previously [30]. The following amplification profile was used: 4 min of initial denaturation at 94°C; 30 cycles of 1 min at 94°C, 1 min at 56°C, and 1 min at 72°C; followed by a final 10 min extension at 72°C. A reaction mixture containing no template DNA was used as a negative control for PCR assays. Reaction products were analyzed using an ABI PRISM 3100-Avant automated DNA sequencer. Each PCR product was analyzed on the sequencer three times and each panel contained at least one sample with a reaction product of known size. Moreover, several samples were sequenced in the automated sequencer and the number of CAG repeats was determined.

### Statistics

The Genepop software (v 4.1.0; http://kimura.univ-montp2.fr/~rousset/Genepop.htm) was used to test the Hardy–Weinberg equilibrium. Kolmogorov–Smirnov tests did not indicate deviation from normality for total aggression scores in either the Hadza or the Datoga sample (*p*s > .10). *t*-tests were used for group comparisons of reports of aggression, age, and number of children. A Mann-Whitney *U*-test was used in the case of the number of *AR* CAG repeats due to significant deviations from normality in both ethnic groups. We used the AMOS extension to IBM SPSS (v. 21.0) for structural equation modeling in order to identify associations of *AR* CAG repeat number, ethnicity and age with the number of children, mediated through BPAQ aggression scores. A test for mediation was carried out in accordance with Baron and Kenny [51], and lower and upper limits of the 95% confidence intervals for standardized regression coefficients were calculated with bootstrapping following Hayes [52].

### Ethical statement

Institutional approvals, including university (Moscow State University Ethics Committee; MSUEC) and local governmental agencies (including Tanzanian Commission for Science and Technology; COSTECH), were obtained prior to conducting this study. All subjects gave their informed, verbal consent prior to participation. Verbal consent was deemed appropriate given the low literacy rates in traditional Hadza and Datoga, and was approved by the MSUEC and the COSTECH.

## Results

Table 1 reports descriptive statistics for age, number of children, BPAQ scores, and *AR* CAG repeats for the Hadza and Datoga samples. There were significant differences between Hadza and Datoga for the BPAQ total score and sub-scale aggression scores, with the Hadza reporting less aggression. The median number of *AR* CAG repeats was greater in the Hadza than in the Datoga (Table 1).

**Table 1:** Descriptive statistics of age, number of children, aggression, and the number of *AR* CAG repeats in Hadza (H) and Datoga (D) men.

		*N*	Min	Max	*Mean*	*SE*	*T*	*df*	*P*
**Age**	**H**	210	17	70	34.76	0.93	0.17	437	0.86
	**D**	229	17	70	34.54	0.86			
**Number of children**	**H**	209	0	14	3.15	0.22	-1.39	387.2	0.17
	**D**	229	0	32	3.72	0.34			
**Physical aggression**	**H**	198	16	42	26.08	0.37	-4.54	382	<0.001
	**D**	186	16	42	28.50	0.38			
**Verbal aggression**	**H**	198	5	25	15.79	0.30	-4.86	381	<0.001
	**D**	185	5	25	17.88	0.31			
**Anger**	**H**	198	8	33	19.58	0.33	-6.80	382	<0.001
	**D**	186	11	34	22.69	0.32			
**Hostility**	**H**	198	10	38	23.25	0.43	-9.53	382	<0.001
	**D**	186	10	40	28.90	0.40			
**Total aggression**	**H**	198	53	125	84.70	1.12	-8.45	381	<0.001
	**D**	185	56	128	97.94	1.09			
***AR* CAG repeat number**	**H**	183	17	30	22*		9684.5^†^	-1.981^‡^	<0.05
	**D**	122	15	31	21*				

Note

*-Median

^†^ - Mann-Whitney *U* statistics

^‡^- *Z* adjusted criterion.

**Fig 1:**
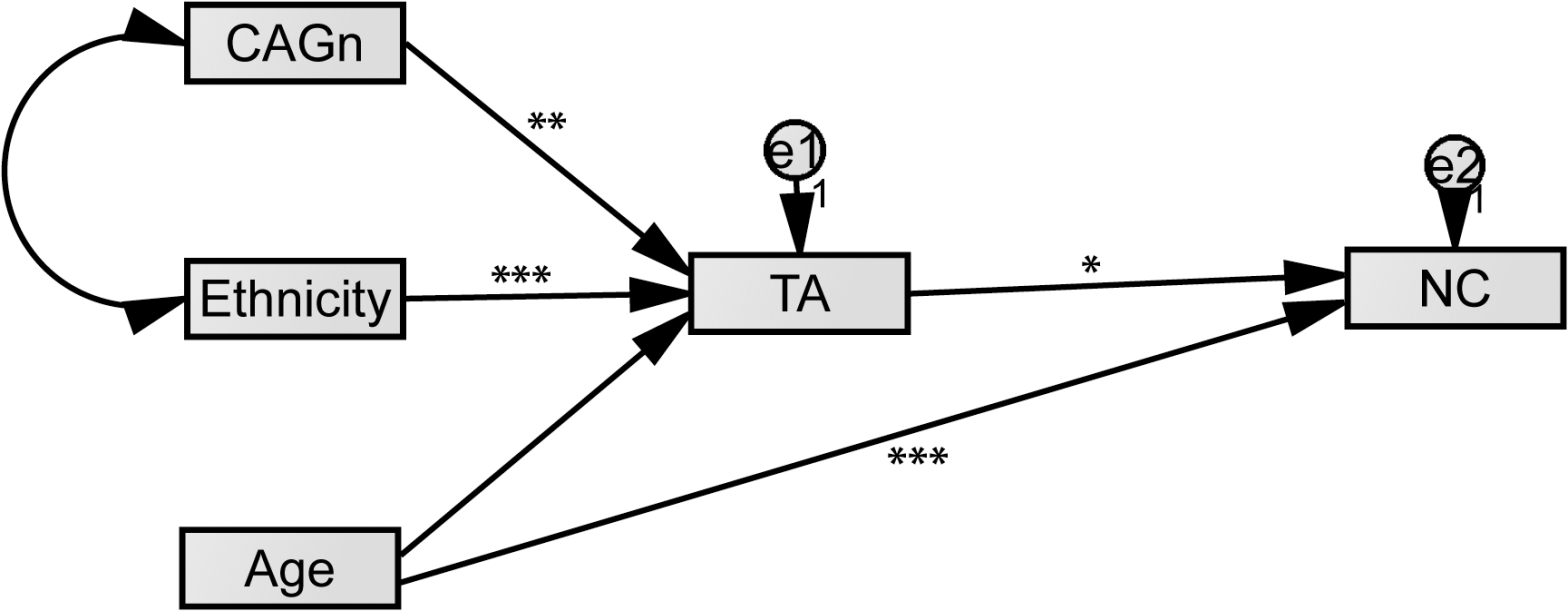
The associations between age group (Age, manifested exogenous variable), number of CAG repeats of *AR* gene (CAGn, manifested exogenous variable), ethnic group (Ethnicity, manifested exogenous variable) and number of children (NC, dependent variable), mediated by total aggression (TA, endogenous mediating variable) scores based on structural equation modeling; asterisks designate significance level: *- 0.01<p≤0.05, **- 0.001<p≤0.01, ***-p<0.001.

We conducted structural equation modeling, with age group, ethnic group, and number of CAG repeats of *AR* gene as exogenous factors predicting number of children through the mediating effect of aggression. Because attitudes toward certain types of aggression may differ between the Hadza and Datoga, we used total aggression scores. Total aggression was negatively associated with CAG repeat number. Age group did not predict aggression (Table 2, Fig 1). Total aggression mediated the association of ethnic group and CAG repeat number (Table 3). The model demonstrated reasonable fit to the data (Chi-square = 9.06, *df* = 4, *p* = .060, Table 4).

**Table 2:** Standardized regression weights according to the model with lower and upper limits of the 95% confidence interval.

Relationship			Estimate	Lower	Upper	P
TA	<—-	Ethnic	.43829	.34106	.52457	.00045
TA	<—-	CAGn	-.14547	-.24464	-.04364	.00823
TA	<—-	Age	-.00048	-.10444	.09905	.97417
NC	<—-	TA	.10534	.02210	.18918	.01121
NC	<—-	Age	.64616	.57154	.70491	.00066

S.E.–standard error of regression weights, C.R.–critical ratio, P–probability value.

**Table 3:** Results of the test for mediation.

Relationship	Direct, without mediation	With Mediation	Indirect
Age—> NC	0.648 (<0.001)	-0.0005 (NS)	NS, no mediation
Ethnic—> NC	0.136 (0.002)	0.438 (<0.001)	0.010, mediation
CAGn—> NC	0.050 (0.246)	-0.145 (0.005)	0.008, mediation

**Table 4:** Goodness of model fit.

Measure of Fitness	
CMIN/DF	2.27 (1.00–3.00)
CFI	0.979 (>0.95)
PCLOSE	0.27 (>0.05)
RMSEA	0.065 (<0.06)

CMIN/DF—chi square/degree of freedom ratio; CFI–Confirmatory Fit Index; RMSEA–the Root Mean Square Error of Approximation; PCLOSE—the p-value for a test of close fit.

**Fig 2:**
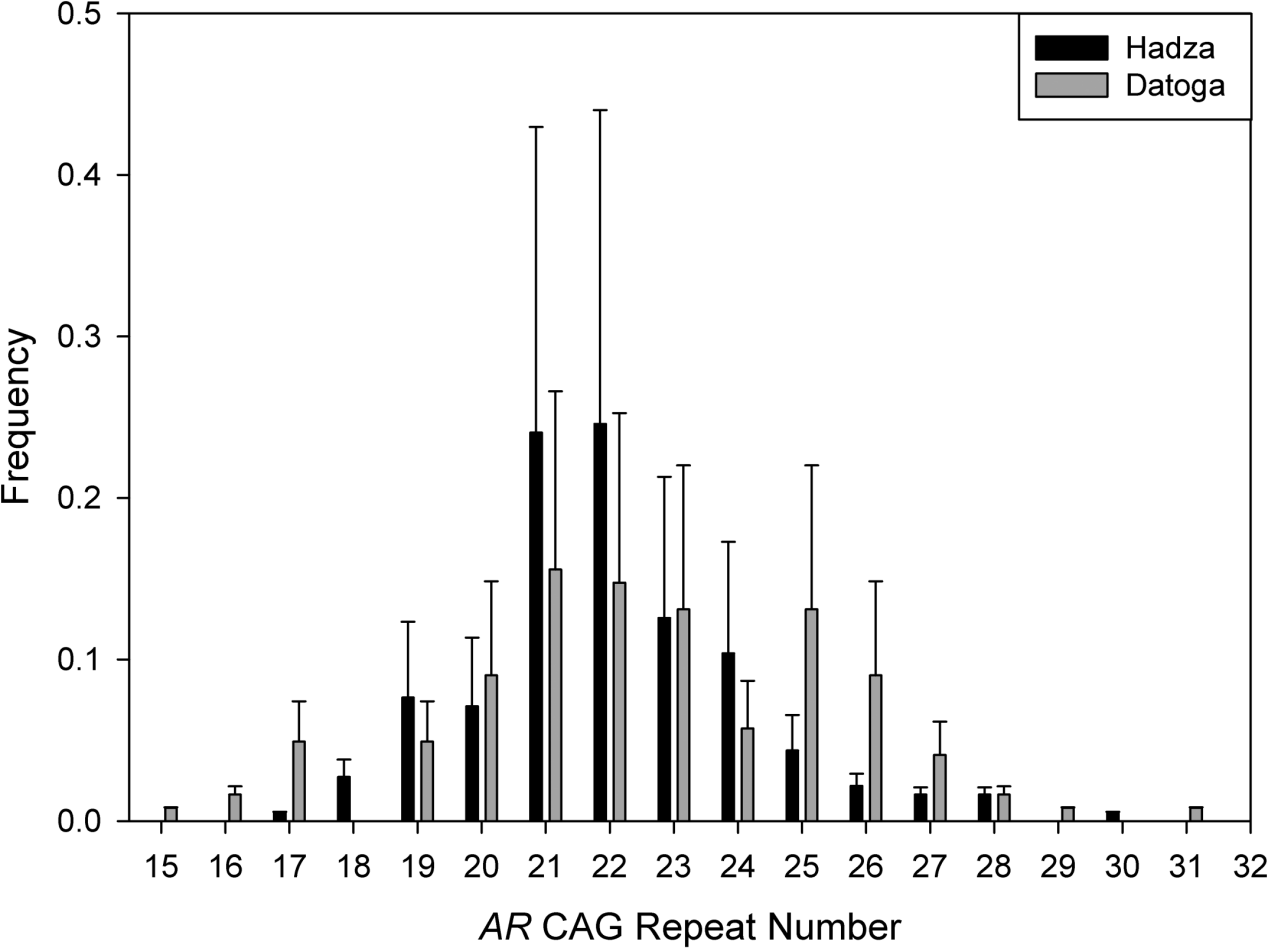
The distributions of allele frequencies at the *AR* CAG locus in Hadza and Datoga men. The x-axis reflects the number of CAG repeats at the *AR* locus. Whisker plots represent 95% confidence intervals.

The frequencies of *AR* CAG repeats for the Hadza and Datoga are shown in Fig 2. Hadza and Datoga men differed in the variability of CAG repeats, with the Datoga showing greater variability at the *AR* locus.

**Fig 3:**
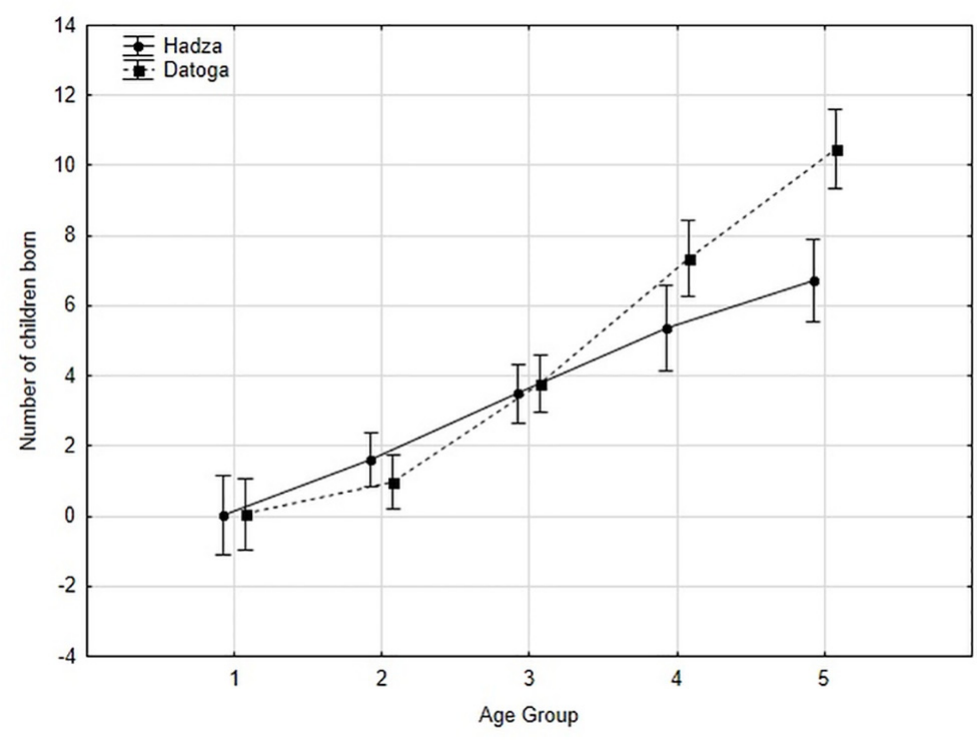
The association between the number of children and fathers’ age in Hadza and Datoga separately represented by five age groups (1 –fathers younger than 20 years of age; 2 –fathers from 20 to younger than 30 years of age; 3 –fathers from 30 to younger than 40 years of age; 4 –fathers from 40 to younger than 50 years of age; 5 –fathers from 50 years of age and older).

Table 5 reports the mean number of children per age group for the Hadza and Datoga. Age groups 5 and 6 (50–59 year olds and 60+ year olds) were combined into one group, as there were only a few participants (*n* = 10 and 12, respectively) in the 60+ group. Older men had more children than younger men. The Hadza men had more children than Datoga men between 20–29 years, whereas a tendency to have more children after the age of 40 in Datoga men was demonstrated (Table 5, Fig 3). Datoga men had more children than Hadza after the age of 50 (Table 5).

**Table 5:** Sample sizes of age groups and mean number of children (± *SE*) per age group in Hadza and Datoga men.

**Age group**	**1**	**2**	**3**	**4**	**5**
Hadza (*n*)	28	57	44	25	26
Datoga (*n*)	20	31	39	17	15
Hadza (*M* ± *SE*)	0.03 ±0.03	1.6 ± 0.2	3.5 ± 0.4	5.4 ± 0.5	6.7 ± 0.7
Datoga (*M* ± *SE*)	0.05 ± 0.04	1.0 ± 0.2	3.8 ± 0.4	7.4 ± 0.8	10.5 ± 1.4
Pairwise comparison of Hadza and Datoga in the number of children, *P* * (see Fig 3)	1.000	0.998	0.999	0.759	0.019

Note: * these are probability values obtained in *post hoc* test using Scheffe’s modification as the most conservative among all others. Age group: 1 = 17–19 years; 2 = 20–29; 3 = 30–39; 4 = 40–49; 5 = 50–60+ years

## Discussion

The main findings of this study confirm the relevance of the challenge hypothesis to humans in natural settings. We demonstrated a positive association between self-reported aggression and reproduction in men. Moreover, we demonstrated a negative association between the CAG *AR* gene repeat number and reproduction, via aggression as mediating factor. We also identified a negative association between aggression and the number of CAG *AR* gene repeats, thus corroborating our previous findings on a smaller sample [29].

Datoga males reported greater aggression than Hadza men–a finding in line with previous reports [29,30]. It is important to mention several striking differences between these two cultures. There is a negative attitude toward aggression among the Hadza but not among the Datoga. In situations of potential aggression, the Hadza prefer to leave [30]. In contrast, aggression is an instrument of social control–both within the family and in outgroup relations in Datoga society. Datoga men are trained to compete with each other and to act aggressively in particular circumstances [30].

Polymorphism of the *AR* CAG repeat number in this study was lower in Hadza men (17–30) than in Datoga men (15–31), consistent with previous findings [29,30]. This greater variability in Datoga than in Hadza men may be due to the difference in sizes of study populations. Given the small current population size in Hadza (about 1000 individuals) [30], and data obtained from genetic analyses, the Hadza may have passed through a genetic bottleneck in recent history [53]. The Datoga population is much larger (between 50000 and 100000 individuals) [40], with no such genetic bottleneck having operated in the recent past. Nevertheless, the degree of genetic variation in both groups fell within the range reported for other African populations, including the Ariaal of Kenya (15–34) [54], as well as other African populations [55].

The structural equation modeling analyses produced several notable results. Our first hypothesis was confirmed: We documented a negative association between the number of CAG repeats in the *AR* gene and total aggression. Our second hypothesis was partly confirmed: Datoga men were more aggressive than Hadza men, but no age effect on aggression was found. Perhaps ethnic differences reveal an effect of social environment, rooted in different population histories and cultural factors.

The current research suggests that the *AR* gene may affect the development and maintenance of masculine traits and male reproduction. That is, along with the association between the number of *AR* CAG repeats and aggression, we may expect the negative correlation of *AR* CAG repeats with general threat potential (e.g., body robustness, physical strength and voice pitch). These qualities may be beneficial in different ways in hunter-gatherers, pastoralists, and horticulturalists, and the modulating role of *AR* in energy allocation under conditions of energy limitation might be addressed in future research. For example, *AR* CAG repeat number may modulate the impact of T on body size and body composition for Ariaal men suffering nutritional deficit [56].

Our findings provide support of our third hypothesis: We identified a positive association between total aggression and the number of children. The negative association of CAG *AR* polymorphism was mediated by aggression.

Our research indicates a difference in the number of children in Hadza and Datoga men achieved after the age of 50. This may be interpreted as differences attributable to different life trajectories and marriage patterns. Beginning in early childhood, boys in the two societies are subjected to different social and environmental pressures (e.g., it is typical for Datoga parents to punish children for misbehavior, while parental violence is much less typical for Hadza parents). Hadza men start reproducing in the early 20s, but their reproductive success later in life is associated with their hunting skills [15]. In the Datoga, men marry later, typically in their 30s. Male status and, consequently, social and reproductive success in the Datoga are positively correlated with fighting abilities and risk-taking in raiding expeditions among younger men, and with wealth, dominance, and social skills among older men. In the Datoga, as in other patrilineal societies, fathers do not invest directly in child care, but children do benefit from their father’s investment in the form of wealth and social protection, as well as various services provided by father’s patrilineal male relatives [56]. In polygynous societies, spending resources on attracting additional wives may be more beneficial [40,57,58]. It would be difficult for some men to invest directly in providing for all their children, given that men with multiple wives can father a considerable number of children, and that households with wives may be located at substantial distance from one another.

The current research includes several limitations. We used data on self-reported number of children, but Hadza and Datoga men sometimes have extramarital sexual affairs and, thus, the actual number of children might be higher. Conversely, it is possible that some children who are related to a particular man were fathered by his wife’s extramarital partner (in the Hadza) or by his brothers or other relatives, as is culturally permissible in the Datoga. Our findings are in concordance with other research, demonstrating that even among the relatively egalitarian Hadza there is selection pressure in favor of more masculine men [59–62]. At the same time, preference for more masculine partners, with greater height and body size, is culturally variable and influenced by the degree of polygyny, local ecology, and other economic and social factors [59–62]. Many Datoga women commented that they would like to avoid taller and larger men as marriage partners, as they may be dangerously violent [44,62]. Only 2% of Hadza women listed large body size as an attractive mate characteristic [63]. Hadza marriages in which the wife is taller than the husband are common, and as frequent as would be expected by chance [64]. We could not exclude the influence of social desirability: attitudes towards aggression are different in Hadza and Datoga, as is tolerance towards potential aggressors. The data on aggression is based on self-ratings, and may be to some extent subjective. Ethological observations in parallel with interviews may be desirable as a future extension of this research to answer this question.

In summary, we suggest that the expression of the *AR* gene may be relevant to aggression and reproduction in men in traditional small-scale societies, where modern methods of contraception are not practiced. We show that aggression, ethnicity, and age predict the number of children sired by men. The number of children sired by men among the Hadza hunter-gatherers and the Datoga semi-nomadic pastoralists differed significantly only for ages 50 older–an effect that may be attributable to polygyny among the Datoga.

## Supporting Information

S1 File Supporting information Buss-Perry_swahili.doc contains translation of the BPAQ in Swahili. (DOC)
