# Supplementary material for: Androgen Receptor Gene Polymorphism, Aggression, and Reproduction in Tanzanian Foragers and Pastoralists
Source: PLoS One. 2015 Aug 20;10(8):e0136208. doi: 10.1371/journal.pone.0136208 (PMC4546275; doi:10.1371/journal.pone.0136208)
Supplement: S1 File — (DOC) [file pone.0136208.s001.doc]

Maswali juu ya Ukatiri (Buss & Perry, 1992) Maelekezo:

Tumia vigezo vitano hapo chini, kuonyesha jinsi vinavyoweza au visivyoweza kuelezea tabia yako. Weka alama katika kisanduku upande wa kulia.

| 1 | 2 | 3 | 4 | 5 |
| --- | --- | --- | --- | --- |

1. **Siyo tabia yangu kabisa**
2. **Kama vile si tabia yangu**
3. **Siyo tofauti sana na tabia yangu lakini si tabia yangu**
4. **Kama vile tabia yangu**
5. **Ni tabia yangu kabisa**

| 1 | Baadhi ya rafiki zangu ufikiri nimepagawa | 1 | 2 | 3 | 4 | 5 |
| --- | --- | --- | --- | --- | --- | --- |
| 2 | Ikinibidi kutumia nguvu ili kujilinda, nitafanya hivyo | 1 | 2 | 3 | 4 | 5 |
| 3 | Wenzangu wakionyesha upendo sana kwangu, najiuliza wanataka nini | 1 | 2 | 3 | 4 | 5 |
| 4 | Nawaeleza marafiki zangu ukweli, pale ninapotofautiana nao | 1 | 2 | 3 | 4 | 5 |
| 5 | Nikichukia nakuwa kama mwendawazimu na naweza kuvunja vitu | 1 | 2 | 3 | 4 | 5 |
| 6 | Siwezi kusaidia kuanzisha majadiriano kama wenzangu wanatofautiana na mimi | 1 | 2 | 3 | 4 | 5 |
| 7 | Najiuliza kwanini wakati mwingine, nakuwa mkali juu ya vitu vingine | 1 | 2 | 3 | 4 | 5 |
| 8 | Inapotokea nikashindwa kuendesha majadiriano ni afadhari achaguliwe mtu mwingine | 1 | 2 | 3 | 4 | 5 |
| 9 | Hata hivyo mimi ni mtu mwenye maamuzi ya haraka | 1 | 2 | 3 | 4 | 5 |
| 10 | Huwa nina wasiwasi na rafiki nisiyemjua vyema | 1 | 2 | 3 | 4 | 5 |
| 11 | Nilishawahujumu wenzangu ninaowajua | 1 | 2 | 3 | 4 | 5 |
| 12 | Nachukia na kumaliza hasira zangu haraka | 1 | 2 | 3 | 4 | 5 |
| 13 | Nikiwa na mda mwingi wa majibizano, naweza kukosana na watu wengine | 1 | 2 | 3 | 4 | 5 |
| 14 | Wenangu wakinichukiza, nawambia ninachofikiri kwao | 1 | 2 | 3 | 4 | 5 |
| 15 | Wakati mwingine najisiki kuwa na wivu sana | 1 | 2 | 3 | 4 | 5 |
| 16 | Huwa sifikirii kuwa siyo vizuri kuwakasirikia wenzangu | 1 | 2 | 3 | 4 | 5 |
| 17 | Mda mwingine nafikiria kwamba sheria ipo nje ya maisha yangu | 1 | 2 | 3 | 4 | 5 |
| 18 | Nina matatizo ya kuzuia hasira | 1 | 2 | 3 | 4 | 5 |
| 19 | Nikiwa na matatizo, naonyesha hasira yangu | 1 | 2 | 3 | 4 | 5 |
| 20 | Wakati mwingine nafikiri wenzangu wananicheka wanapokuwa nyuma yangu | 1 | 2 | 3 | 4 | 5 |
| 21 | Mara nyingine najikuta; nikitofautiana na wenzangu | 1 | 2 | 3 | 4 | 5 |
| 22 | Mwenzangu akinipiga lazima nimrudishie | 1 | 2 | 3 | 4 | 5 |
| 23 | Wakati mwingine najiona kama bomu linalosubili kulipuka | 1 | 2 | 3 | 4 | 5 |
| 24 | Watu wengine maranyingi wanatafuta ufa wapate pa kuanzia | 1 | 2 | 3 | 4 | 5 |
| 25 | Kuna wenzangu ambao wamesababisha tupigane | 1 | 2 | 3 | 4 | 5 |
| 26 | Najua kuwa hawa “fafiki zangu” wananizungumuzia nikiwa sipo | 1 | 2 | 3 | 4 | 5 |
| 27 | Rafiki zangu wanasema kuwa mimi ni mbishi sana | 1 | 2 | 3 | 4 | 5 |
| 28 | Maranyingine hujitoa bila kuwepo na sababu ya msingi | 1 | 2 | 3 | 4 | 5 |
| 29 | Najiingiza kwenye ugonvi haraka bila subira | 1 | 2 | 3 | 4 | 5 |
